# Questionnaire survey on the clinical application of Hall technique preformed metal crowns in caries of primary molars in China

**DOI:** 10.3389/froh.2024.1513840

**Published:** 2025-01-13

**Authors:** Junhui Wang, Baize Zhang, Yujiang Chen, Lulu Wang, Yang Du, Xin Ge, Fen Liu, Jing Gong, Xiaojing Wang

**Affiliations:** ^1^ State Key Laboratory of Oral & Maxillofacial Reconstruction and Regeneration, National Clinical Research Center for Oral Diseases, Shaanxi Clinical Research Center for Oral Diseases, Department of Pediatric Dentistry, School of Stomatology, The Fourth Military Medical University, Xi'an, China; ^2^ Department of Stomatology, Xi'an People’s Hospital (Xi'an Fourth Hospital), Xi'an, China; ^3^ Key Laboratory of Shaanxi Province for Craniofacial Precision Medical Research, College of Stomatology, Xi’an Jiaotong University, Xi’an, China; ^4^ Department of Pediatric Dentistry, Affiliated Stomatology Hospital of Xi’an Jiaotong University, Xi’an, China

**Keywords:** children, primary molars, preformed metal crowns, Hall technique, questionnaire survey

## Abstract

**Objective:**

To analyze the clinical application and limiting factors of the Hall technique (HT) preformed metal crowns in caries of primary molars and to provide reference for its clinical application based on a questionnaire survey.

**Materials and methods:**

From June to December 2022, a questionnaire was distributed to pediatric dentists through the WeChat platform using “Questionnaire Star” to analyze the clinical application and influencing factors of the HT preformed metal crowns in caries of primary molars.

**Results:**

A total of 700 survey questionnaires were distributed, and 650 valid questionnaires were recovered, with a effectiveness rate of 92.86%. In terms of the awareness level of the HT, only 11.08% of pediatric dentists fully understood, while 19.85% of those were completely unaware; In terms of the understanding methods, classes study accounted for 47.60%, and 28.79% of pediatric dentists understood through literature; In terms of the application of the HT, 46.15% of pediatric dentists had applied it, only 11.23% had frequently applied it, and 53.85% had never applied it; In terms of the usage time, the highest proportion was between 1 and 3 years, accounting for 40.33%, and only 9.67% were over 5 years; In terms of the choosing of indications, only 26.62% of pediatric dentists could select indications correctly; Regarding the choice of treatment methods for children who do not cooperate, only 19.54% of pediatric dentists chose the HT. The education background and nature of the workplace had a statistically significant difference in the application of the HT, the number of years of application, the selection of indications, and the choice of treatment methods for children who do not cooperate.

**Conclusions:**

The survey showed that the awareness level and application frequency of the HT were relatively low in China. The understanding methods mainly learned through classes study.

**Clinical relevance:**

It is necessary to further promote the development of continuing education projects with the theme of clinical application of the HT and the guidance of clinical operation standards, in order to promote the clinical popularization of it in China.

## Introduction

Dental caries is considered to be the single most common chronic childhood disease, and its prevalence is thought to have increased recently in children 2–5 years of age worldwide, making this age group a global priority action area (1). The Fourth Epidemiological survey on oral health showed that the caries rate among 5-year-old children was 70.9%, with an increase of 5.8% compared to the third survey in 2005 (66%) in China (2). In developed countries such as Italy, New Zealand, and the United States, the caries rates of deciduous teeth were 14.4%, 15.8%, and 23% (3–5), respectively, which were significantly lower than those in China; In developing countries such as El Salvador, the caries rate of deciduous teeth (58%) (6) was also lower than in China. Compared with the high prevalence of primary dental caries, the treatment proportion of 5-year-old children in China was only 4.1%, which was still at a low level, although it had increased compared to the third survey (2.8%) (7).

Due to the young age, poor tolerance, and limited self-control, children often exhibit nervousness, fear and even avoidance or refusal in the treatment of oral diseases. This manifestation not only affects the treatment, but also can extend to adults. About 20.1% of children aged 4–6 are unable to cooperate in completing the normal oral diagnosis and process of treatment in China (8), and 85% of dental phobia of adults occurs during childhood (9). Therefore, how to alleviate nervousness and fear during the treatment process is particularly crucial.

The conventional treatment of deciduous tooth caries is removing decay and preparing cavities with dental drills, followed by resin restorations. Traditional high speed dental drills are widely used for rapid removal of decayed enamel and dentin, but when preparing cavities, dental drills are noisy, vibrate and may remove too much normal tooth tissue during caries removal. Meanwhile, when dental caries is removed by traditional mechanical rotary devices, the pulp is frequently subjected to pressure and heat effects, which usually cause pain and pulpal damage (10). It is difficult to ensure the quality of treatment, due to the young age and low level of cooperation of pediatric patients, coupled with the drawbacks of traditional high speed dental drills.

In 1988, Straffon et al. (11) proposed the theory that the development process of strictly sealed dentin caries would be slow or stagnant. Ricketts compared the effects of complete and partial removal of decayed tissues on the dental pulp, demonstrating that partial removal of decayed tissues had the greatest benefit for the health of pulp (12). The Hall technique (HT) was proposed based on the above theory. The HT is a restoration method which does not require tooth preparation, local anesthesia, and caries removal, but simply uses preformed metal crowns to completely isolate the teeth from the oral cavity (13, 14). Therefore, it will not cause fear, anxiety, which is more suitable for pediatric patients, compared with traditional mechanical rotary devices. In a survey of pediatric dentists, the HT was preferred for treating asymptomatic primary molars caries in children with dental anxiety (15), and the same results were obtained in a survey of pediatric dentists across Europe (16). Although the HT has been widely used in pediatric dentistry in such as European countries, it was applied relatively late in China.

The aim of this study is to use a questionnaire survey to understand the application and influencing factors of the HT, in order to provide reference for its application in China.

## Materials and methods

### Survey methods

Firstly, by reviewing the relevant literature of “stainless steel crown of primary molar”, “preformed metal crown of primary molar”, “repairing method of primary molar” and “Hall technique” in CNKI (China National Knowledge Infrastructure Database) database and VIP (Wei Pu) full-text database, we summarized the problems in the clinical application of the HT in primary molar, determined the content of the questionnaire and compiled it. The questionnaire included two aspects: one part was the demographic data of pediatric dentists, including gender, age, working experience, educational background and the working place; the second part was the clinical application and influencing factors of the HT. Before completing the questionnaire, the questionnaire survey personnel received theoretical and technical training and completed the standard consistency test (Kappa values of two pediatric dentists were 0.97 and 0.96, respectively, and the Kappa values between the two examiners were 0.95). This study protocol was approved by the institutional research ethics committee at the Stomatological Hospital of the Fourth Military Medical University, China (Approval No. IRB-REV-2021124). All methods were performed in accordance with the Declaration of Helsinki. All the respondents had given informed consent, voluntarily participated in the survey and filled out the questionnaire independently. If there are any questions in the filling process, the questionnaire designer should be responsible for answering them.

Prior to the formal survey, we conducted a preliminary survey and improved the survey questionnaire. At the same time, we also verified the reliability and validity of the questionnaire, both of which met the requirements of the questionnaire survey. 105 questionnaires were distributed, and 100 valid questionnaires were collected, and the number of questionnaires needed was estimated. According to the preliminary survey results, the awareness rate of the HT was 40% (40/100). Calculate formula *N* = *Uα*^2^*P*(1 - *P*) ÷ *d*^2^, (*Uα* is the bound value at a certain confidence level, *P* is the probability value, and d is the sampling error rate). This survey used a 95% confidence level, therefore *Uα* = 1.96. To ensure the accuracy of the survey, the error rate (maximum allowable absolute error) was set to 4%, and an effective sample size of 577 was calculated. Stratification factors and no response rate (calculated at 20%) were fully considered, resulting in a total sample size of 693.

### Survey subjects

From June to December 2022, an electronic questionnaire was written through “QuestionStar” and distributed to 700 pediatric dentists through WeChat platform. The questionnaire was filled online to understand the application and influencing factors of the HT in China. All surveyed dentists were informed of the survey purpose and voluntarily filled the survey questionnaire.

### Statistical analysis

Building a database with Excel software, SPSS 22.0 software was used to perform statistical analysis. The data were described by frequency and percentage, *χ*^2^ test was used to analyze the understanding, clinical application and influencing factors of the HT, and two-sided *P* < 0.05 was considered as statistically significant.

## Results

### The recovery of the questionnaires

In this survey, 700 questionnaires were distributed, and 678 questionnaires were recovered, the recovered rate was 96.86%. 28 questionnaires were excluded due to information omissions, incomplete options and contradictory answers, and a total of 650 valid questionnaires were collected, the rate of the effective questionnaires was 92.86%.

### Knowledge of Hall technique among pediatric dentists

In terms of the awareness level of the HT, only 11.08% of pediatric dentists fully understood, while 19.85% of those completely unknown. Among them, there was no statistically significant difference in the impact of gender on the awareness level of the HT, while there were statistically significant differences in the impact of age, working experience, educational background, and the nature of the workplace on the awareness level. The awareness rate of pediatric dentists over 50 years old, working for more than 20 years, doctoral graduation and specialized dental hospital were significantly higher than those of the other groups (Table 1).

**Table 1:** Knowledge of Hall technique among pediatric dentists (*n*, %).

Influencing factor	Number	Cognitive level				*χ* ^2^	*P*
		Completely unknown	A little understanding	General understanding	Fully understand		
	650	129 (19.85)	246 (37.85)	203 (31.23)	72 (11.08)		
Gender						1.701	0.637
Male	316 (48.62)	58 (18.35)	120 (37.97)	105 (33.23)	33 (10.45)		
Female	334 (51.38)	71 (21.26)	126 (37.72)	98 (29.34)	39 (11.68)		
Age						25.72	0.002*
22–30	181 (27.85)	49 (27.07)	72 (39.78)	47 (25.97)	13 (7.18)		
30–40	186 (28.62)	40 (21.51)	65 (34.95)	62 (33.33)	19 (10.21)		
40–50	188 (28.92)	30 (15.96)	79 (42.02)	58 (30.85)	21 (11.17)		
>50	95 (14.61)	10 (10.53)	30 (31.58)	36 (37.89)	19 (20.00)		
working experience						27.65	0.001*
0–3 years	167 (25.70)	48 (28.74)	63 (37.72)	46 (27.54)	10 (6.00)		
3–10 years	192 (29.54)	40 (20.83)	73 (38.02)	59 (30.73)	20 (10.42)		
10–20 years	202 (31.08)	34 (16.83)	80 (39.60)	64 (31.68)	24 (11.89)		
>20 years	89 (13.68)	7 (7.87)	30 (33.71)	34 (38.20)	18 (20.22)		
Education						47.46	<0.0001*
Junior college graduate	98 (15.08)	37 (37.76)	30 (30.61)	26 (26.53)	5 (7.14)		
Undergraduate	189 (29.08)	44 (23.28)	79 (41.80)	49 (25.93)	17 (8.99)		
Master	281 (43.23)	39 (13.88)	114 (40.57)	96 (34.16)	32 (11.39)		
Doctor	82 (12.62)	9 (10.98)	23 (28.05)	32 (39.02)	18 (21.95)		
Nature of workplace						95.17	<0.0001*
Specialized dental hospital	207 (31.85)	30 (14.49)	40 (19.32)	102 (49.28)	35 (16.91)		
General hospital	278 (42.77)	52 (18.71)	117 (42.08)	84(30.22)	25(8.99)		
Clinic	165(25.38)	47(28.49)	89(53.94)	17(10.30)	12(7.73)		

* Statistically signifcant diference (chi-square test).

### Understanding modes of Hall technique by pediatric dentists

In terms of the understanding modes of the HT, course teaching accounted for 47.60% and literature review accounted for 28.79%. There were no significant differences in the impact of gender, age, working experience, educational background, and the nature of the workplace on the understanding modes of the HT (Table 2).

**Table 2:** Understanding modes of Hall technique by pediatric dentists (*n*, %).

Influencing factor	Number	Understanding modes				*χ* ^2^	*P*
		Class teaching	Literature review	Unintentional understanding	Others		
	521	248 (47.60)	150 (28.79)	73 (14.01)	50 (9.60)		
Gender						1.796	0.616
Male	258 (49.52)	117 (45.35)	81 (31.40)	36 (13.95)	24 (9.30)		
Female	263 (50.48)	131 (49.81)	69 (26.24)	37 (14.07)	26 (9.88)		
Age						3.746	0.927
22–30	132 (25.34)	58 (43.94)	37 (28.03)	22 (16.67)	15 (11.36)		
30–40	146 (28.02)	70 (47.94)	41 (28.08)	21 (14.38)	14 (9.60)		
40–50	158 (30.33)	78 (49.37)	45 (28.48)	19 (12.03)	16 (10.13)		
>50	85 (16.31)	42 (49.41)	27 (31.76)	11 (12.94)	5 (5.82)		
Working experience						5.667	0.773
0–3 years	119 (22.84)	56 (47.06)	33 (27.73)	19 (15.97)	11 (9.24)		
3–10 years	152 (29.17)	72 (47.37)	44 (28.95)	21 (13.82)	15 (9.87)		
10–20 years	168 (32.25)	80 (47.62)	45 (28.57)	24 (14.29)	19 (11.31)		
>20 years	82 (15.74)	40 (48.78)	28 (34.15)	9 (10.98)	5 (6.10)		
Education						3.549	0.939
Junior college graduate	61 (11.71)	32 (52.46)	15 (24.59)	9 (14.75)	5 (8.20)		
Undergraduate	145 (27.83)	72 (49.66)	45 (28.39)	16 (11.03)	12 (8.28)		
Master	242 (46.45)	109 (45.04)	70 (28.93)	38 (15.70)	25 (10.33)		
Doctor	73 (14.01)	35 (47.95)	20 (27.40)	10 (13.70)	8 (10.95)		
Nature of workplace						9.145	0.166
Specialized dental hospital	177 (33.97)	69 (38.98)	56 (31.64)	31 (17.51)	21 (11.86)		
General hospital	226 (43.38)	115 (50.88)	64 (28.32)	28(12.40)	19(8.41)		
Clinic	118(22.65)	64(54.24)	30(25.42)	14(11.86)	10(8.47)		

### Application of Hall technique by pediatric dentists

In terms of the application of the HT, 46.15% of pediatric dentists had applied, while 53.85% of those had never applied, frequent applications only accounting for 11.23%. Gender, age, and working experience had no statistically significant differences in the application of the HT, while educational background and the nature of the workplace have statistically significant differences in the application (Table 3).

**Table 3:** Application of Hall technique by pediatric dentists (*n*, %).

Influencing factor	Number	Application situation				*χ* ^2^	*P*
		Often	Sometimes	Rarely	Never		
	650	73 (11.23)	100 (15.38)	127 (19.54)	350 (53.85)		
Gender						1.341	0.716
Male	316 (48.62)	34 (10.76)	53 (16.77)	58 (18.35)	171 (54.12)		
Female	334 (51.38)	39 (11.68)	47 (14.07)	69 (20.66)	179 (53.59)		
Age						12.64	0.179
22–30	181 (27.85)	15 (8.29)	27 (14.92)	34 (18.78)	105 (58.01)		
30–40	186 (28.62)	28 (15.05)	30 (16.13)	35 (18.82)	93 (50.00)		
40–50	188 (28.92)	25 (13.30)	30 (15.96)	32 (17.02)	101 (53.72)		
>50	95 (14.61)	5 (5.26)	13 (13.68)	26 (27.37)	51 (53.68)		
Working Experience						10.28	0.328
0–3 years	167 (25.70)	13 (7.78)	25 (14.97)	36 (21.56)	93 (55.69)		
3–10 years	192 (29.54)	29 (15.10)	30 (15.63)	36 (18.75)	97 (50.52)		
10–20 years	202 (31.08)	26 (12.87)	33 (16.34)	34 (16.83)	109 (53.96)		
>20 years	89 (13.68)	5 (5.61)	12 (13.48)	21 (23.60)	51 (57.31)		
Education						19.78	0.019*
Junior college graduate	98 (15.08)	7 (7.14)	11 (11.22)	20 (20.41)	60 (61.22)		
Undergraduate	189 (29.08)	18 (9.52)	22 (11.64)	37 (19.57)	112 (59.26)		
Master	281 (43.23)	34 (12.10)	48 (17.08)	62 (22.06)	137 (48.75)		
Doctor	82 (12.62)	14 (17.07)	19 (23.17)	8 (9.76)	41 (50.00)		
Nature of workplace						21.80	0.001*
Specialized dental hospital	207 (31.85)	34 (16.43)	37 (17.87)	41 (19.81)	95 (45.89)		
General hospital	278 (42.77)	30 (10.79)	48 (17.27)	50(17.99)	150(53.95)		
Clinic	165(25.38)	9(5.45)	15(9.09)	36(21.82)	105(63.64)		

* Statistically signifcant diference (chi-square test).

### Years of application of Hall technique by pediatric dentists

In terms of the application period of the HT, it accounted for 40.33% between 1 and 3 years, while it only accounted for 9.67% over 5 years by pediatric dentists. Gender, age, and working experience had no statistically significant differences in the application period of the HT, while educational background and the nature of the workplace had statistically significant differences in the application period (Table 4).

**Table 4:** Years of application of Hall technique by pediatric dentists (*n*, %).

Influencing factor	Number	Application period				*χ* ^2^	*P*
		>5 years	3–5 years	1–3 years	<1 years		
	300	29 (9.67)	95 (31.67)	121 (40.33)	55 (18.33)		
Gender						1.880	0.598
Male	145 (48.33)	12 (8.28)	51 (35.18)	56 (38.62)	26 (17.93)		
Female	155 (51.67)	17 (10.97)	44 (28.39)	65 (41.94)	29 (18.70)		
Age						7.242	0.612
22–30	76 (25.33)	6 (7.89)	20 (26.32)	32 (42.11)	18 (23.68)		
30–40	93 (31.00)	8 (8.60)	26 (27.96)	40 (43.01)	19 (20.43)		
40–50	87 (29.00)	9 (10.34)	32 (36.78)	33 (37.93)	13 (14.95)		
>50	44 (14.67)	6 (14.63)	17 (41.46)	16 (36.36)	5 (11.35)		
Working experience						10.41	0.318
0–3 years	74 (24.67)	5 (6.76)	18 (24.32)	31 (41.89)	20 (27.03)		
3–10 years	95 (31.67)	9 (9.47)	27 (28.42)	41 (43.16)	18 (18.95)		
10–20 years	93 (31.00)	10 (10.75)	35 (37.63)	35 (37.63)	13 (13.99)		
>20 years	38 (12.66)	5 (13.16)	15 (39.47)	14 (36.84)	4 (10.52)		
Education						18.98	0.025*
Junior college graduate	38 (12.66)	3 (7.89)	9 (23.68)	12 (31.58)	14 (36.85)		
Undergraduate	77 (25.67)	7 (9.10)	18 (23.37)	33 (42.86)	19 (24.67)		
Master	144 (48.00)	14 (9.72)	51 (35.42)	61 (42.36)	18 (12.50)		
Doctor	41 (13.67)	5 (12.20)	17 (41.46)	15 (36.59)	4 (9.75)		
Nature of workplace						16.54	0.011*
Specialized dental hospital	112 (37.33)	14 (12.50)	46 (41.07)	42 (37.50)	10 (8.93)		
General hospital	128 (42.67)	12 (9.38)	34 (26.56)	52(40.63)	30(23.43)		
Clinic	60(20.00)	3(5.00)	15(0.25)	27(0.45)	15(0.25)		

* Statistically signifcant diference (chi-square test).

### Selection of indications for Hall technique by pediatric dentists

In the selection of indications for the HT, only 26.62% of pediatric dentists were able to make the right choices. Gender, age, and work experience had no statistically significant differences in the selection of indications, while educational background and the nature of the workplace had statistically significant differences in the selection of indications (Table 5).

**Table 5:** Indications for Hall technique by pediatric dentists (*n*, %).

Influencing factor	Number	Indications				*χ* ^2^	*P*
		Shallow caries and middle caries	Shallow caries, middle caries and deep caries	Shallow caries, middle caries and enamel hypoplasia	Shallow caries, middle caries, deep caries and enamel hypoplasia		
	650	49 (7.54)	150 (23.07)	173 (26.62)	278 (42.77)		
Gender						2.489	0.477
Male	316 (48.62)	23 (7.28)	68 (21.51)	80 (25.32)	145 (45.89)		
Female	334 (51.38)	26 (7.78)	82 (24.56)	93 (27.84)	133 (39.82)		
Age						9.759	0.370
22–30	181 (27.85)	14 (7.73)	50 (27.62)	40 (22.10)	77 (42.54)		
30–40	186 (28.62)	16 (8.60)	46 (24.73)	55 (29.57)	69 (37.10)		
40–50	188 (28.92)	13 (6.91)	38 (20.21)	53 (28.19)	84 (44.68)		
>50	95 (14.61)	6 (6.32)	16 (16.84)	25 (26.32)	48 (50.52)		
Working experience						9.516	0.391
0–3 years	167 (25.70)	13 (7.18)	48 (28.74)	37 (22.16)	69 (41.32)		
3–10 years	192 (29.54)	17 (8.85)	46 (23.96)	56 (29.17)	73 (38.02)		
10–20 years	202 (31.08)	14 (6.93)	40 (19.80)	56 (27.72)	92 (45.55)		
>20 years	89 (13.68)	5 (5.62)	16 (17.98)	24 (26.97)	44 (49.43)		
Education						22.84	0.007*
Junior college graduate	98 (15.08)	9 (9.18)	30 (30.61)	16 (16.33)	43 (43.88)		
Undergraduate	189 (29.08)	16 (8.47)	45 (23.81)	40 (21.16)	88 (46.56)		
Master	281 (43.23)	19 (6.76)	65 (23.13)	84 (29.89)	113 (40.22)		
Doctor	82 (12.62)	5 (6.10)	10 (12.19)	33 (40.24)	34 (41.46)		
Nature of workplace						84.73	<0.001*
Specialized dental hospital	207 (31.85)	10 (4.83)	30 (14.49)	100 (48.31)	67 (32.37)		
General hospital	278 (42.77)	21 (7.55)	70 (25.18)	60 (21.58)	127(45.69)		
Clinic	165(25.38)	18(10.91)	50(30.30)	13(7.88)	84(50.91)		

* Statistically signifcant diference (chi-square test).

### Treatment methods for no cooperation children by pediatric dentists

Regarding the choice of treatment methods for children who did not cooperate, only 19.54% of pediatric dentists choose the HT. Gender, age, work experience and educational background had no statistically significant differences in the selection of treatment methods for uncooperative children, while the nature of the workplace had statistically significant differences in the selection of treatment methods (Table 6).

**Table 6:** Treatment methods for no cooperationchildren by pediatric dentists (*n*, %).

Influencing factor	Number	Treatment methods				*χ* ^2^	*P*
		Hall technique	General anesthesia	Treatment after cooperation	Other		
	650	127 (19.54)	238 (36.61)	225 (34.62)	60 (9.23)		
Gender						3.073	0.380
Male	316 (48.62)	60 (18.99)	125 (39.56)	106 (33.54)	25 (7.91)		
Female	334 (51.38)	67 (20.06)	113 (33.83)	119 (35.63)	35 (10.48)		
Age						9.139	0.425
22–30	181 (27.85)	30 (16.57)	59 (32.60)	70 (38.67)	22 (12.16)		
30–40	186 (28.62)	37 (19.89)	69 (37.10)	60 (32.26)	20 (10.75)		
40–50	188 (28.92)	40 (21.28)	75 (39.89)	60 (31.91)	13 (6.92)		
>50	95 (14.61)	20 (21.05)	35 (36.84)	35 (36.84)	5 (5.27)		
Working experience						9.027	0.435
0–3 years	167 (25.70)	29 (17.36)	49 (29.34)	66 (39.52)	18 (10.78)		
3–10 years	192 (29.54)	33 (17.19)	71 (36.98)	70 (36.46)	18 (9.37)		
10–20 years	202 (31.08)	46 (22.77)	79 (39.11)	59 (29.21)	18 (8.91)		
>20 years	89 (13.68)	19 (21.35)	34 (38.20)	30 (33.71)	6 (6.74)		
Education						16.71	0.054
Junior college graduate	98 (15.08)	14 (14.29)	29 (29.59)	42 (42.86)	13 (13.26)		
Undergraduate	189 (29.08)	32 (16.93)	61 (32.28)	76 (40.21)	20 (10.58)		
Master	281 (43.23)	60 (21.35)	112 (39.86)	86 (30.60)	23 (8.19)		
Doctor	82 (12.62)	21 (25.61)	36 (43.90)	21 (25.61)	4 (4.88)		
Nature of workplace						19.33	0.023*
Specialized dental hospital	207 (31.85)	49 (23.67)	85 (41.06)	61 (29.47)	12 (5.80)		
General hospital	278 (42.77)	55 (19.78)	104 (37.41)	94(33.81)	25(8.99)		
Clinic	165(25.38)	23(13.93)	49(29.70)	70(42.42)	23(13.95)		

* Statistically signifcant diference (chi-square test).

## Discussion

Our questionnaire conducted a cross-sectional study to analyze the awareness level and usage of the HT among pediatric dentists in China, including specialized pediatric dentists and general dentists, as well as the influencing factors that limited the clinical application of the HT. The HT has been widely used in Australia, Belgium, Brazil, Chile, Germany (17), India, the Netherlands, New Zealand, the United Arab Emirates (18), the United Kingdom (19), and the United States, and has been taught to undergraduate and graduate students in the UK, New Zealand, the United States, Australia, India, as well as some South American and Middle Eastern countries (14). However, the application of the HT in teaching and clinical practice was relatively late in China. Based on this situation, our study used a questionnaire survey to understand the application and influencing factors of the HT in China, in order to provide reference for its application in China.

In 2011, Dean et al. (20) found that the awareness rate of the HT in Scotland was 86%, with a clinical application rate of 48%. In terms of awareness, literature reading accounted for 53%, and the HT had also been widely taught in university courses. In 2018, according to a survey by Roberts et al., 96% of respondents in the UK had used the HT, 60% of respondents had used the HT for over 5 years, and over 90% respondents believed that the HT was suitable for undergraduate teaching, general practice, postgraduate training and specialist practice (16). In 2020, Hussein et al. (21) conducted a global survey, which showed that the awareness rate of the HT was 92.32%, while the application rate of the HT was only 50.6%. The limiting factors were the lack of unified standards and standardized training. Similarly, Crystal et al. (22) found that the teaching rate of the HT in the United States was 90.2%, and the clinical application rate was 69.1%. In 2021, Ezzeldin et al. (23) conducted a survey in the eastern region of Saudi Arabia, which showed that the awareness rate of the HT was 54.9% among dental experts, 55.5% among graduate students, and only 42.7% among current students. The application rate of the HT was 28.2% for dental experts, 18.0% for graduate students, and only 17.7% for current students. Our survey showed that the awareness rate of the HT was significantly lower than that of Dean and Hussein, and slightly lower than Ezzeldin's survey results. The application rate of the HT was significantly lower than Roberts, slightly lower than Dean and Hussein, and higher than Ezzeldin's survey results. In terms of the understanding methods of the HT, our survey showed that it was mainly focused on learning through classes study, while the results of Dean and Yasmi's mainly relied on university teaching. In 2023, Lai (24) conducted a questionnaire survey in the East China, focusing on investigating the perception and use of the HT among students engaging in demographic identity. The survey showed that the usage rate of the HT was 40.2%. Most dentists who had not used the HT were concerned about complications such as pulp inflammation or necrosis after applying the HT. Our survey shows that the awareness rate of the HT was 80.15%, lower than the 91.4% reported in the survey of Lai, and the usage rate our survey was 47.60%, slightly higher than the 40.2% reported in the survey of Lai. The number of our research subjects was 650 dentists, which was twice as much as Lai. At the same time, we also extended the direction of the investigation, setting questions to understand the reasons for limiting the clinical application and the selection of indications of the HT.

The clinical application rate of the HT was relatively low, mainly due to the problem of bite opening. In 2010, Van der Zee's clinical study of 58 pediatric patients found that the HT restored normal occlusion within 2–4 weeks after repairing (25). In 2020, Abu Serdaneh et al. (26) studied the effect of the HT on masseter muscle activity in 12 children. The study showed that after the HT, the masseter muscle activity of children was affected to a certain extent after 2 and 6 weeks. However, due to the relatively small sample size, further validation was required through large sample size clinical studies.

With the increasing number of investigations, there are also more and more clinical studies on the HT. In 2006, Innes et al. (13) published a clinical randomized controlled trial on the the HT. In a 23 month clinical randomized controlled trial, a total of 264 caries teeth were included in the trial. The results showed that 89% of the teeth had no apparent discomfort up to mild, compared to 78% in the control group. The rate of major failure in the HT group was 2%, while the control group was 15%. The rate of minor failure in the HT group was 5%, while the control group was 46%. The success rate of the HT group (93%) was significantly higher than the control group (39%) (13). In 2019, Midani et al. (17) conducted a retrospective study on 181 primary molars that underwent the HT restoration from 2011 to 2017, the results showed that the success rate was 92.3% with an average follow-up time of 22 months. In New Zealand, the research of Boyd (27) showed that the success rate of the HT was 89% at 12 months, and 85% at 24 months. In UAE, the retrospectively study of Binladen (18) showed that the success rate of the HT was 97.6%, significantly higher than the control group's success rate of 93.5% after 24 months. In 2023, The review of Chua et al. (28) showed that the success rate of the HT and traditional metal prefabricated crown restoration method in 12 and 24 months were both over 85%, with no statistically significant difference, but the HT had a short treatment time, low cost-effectiveness and high acceptance among parents.

At the same time, our survey showed that the age, working experience, educational background, and nature of workplace had a statistically significant impact on the level of awareness level. Educational background and nature of workplace had a statistically significant impact on the application, the years of application, the selection of indications of the HT and the choice of treatment methods for children who did not cooperate. There were two main reasons for the above situation. Firstly, preformed metal crown was first proposed in the 1950s, and the HT began to be applied abroad in the 1990s. Preformed metal crowns were applied in the early 21st century in China, the application of the HT was also relatively late. Most clinical dentists had limited understanding about the HT. Secondly, due to the fact that the HT had not yet been reflected in university teaching courses, vocational and undergraduate students had little understanding of it (27). The relatively high awareness rate of graduate students was due to their exposure to public courses and classes study. The relatively high awareness rate of specialized dental hospitals and comprehensive dental departments were that there were relatively more opportunities for continuing teaching, learning and there were also relatively more opportunities for exposure and understanding.

The awareness level and clinical application frequency of the HT in China were relatively low, and there was still a significant gap compared to the developed countries. Therefore, pediatric dentists should master the clinical indications of the HT, strict control of the clinical operation process, further effective and standardized to promote the application of the HT, thereby improving the quality of repair and promoting the clinical popularization of the HT.

## Conclusions

In China, the awareness level and application frequency of the HT in the treatment of caries in children were relatively low and the proportion of correctly selected indications was also relatively low. It is necessary to further promote the development of continuing education projects with the theme of clinical application of the HT and the guidance of clinical operation standards, in order to promote the clinical popularization of the HT.

## Data Availability

The raw data supporting the conclusions of this article will be made available by the authors, without undue reservation.
